# The Potential Role of Circulating MicroRNAs in Male Rat Infertility Treated with *Kaempferia parviflora*

**DOI:** 10.1155/2021/9622494

**Published:** 2021-12-17

**Authors:** Hadeel A. Al-Rawaf, Sami A. Gabr, Ahmad H. Alghadir

**Affiliations:** ^1^Department of Clinical Laboratory Sciences, College of Applied Medical Sciences, King Saud University, Riyadh, Saudi Arabia; ^2^Department of Rehabilitation Sciences, College of Applied Medical Sciences, King Saud University, Riyadh, Saudi Arabia

## Abstract

**Background:**

Therapeutic strategies based on herbal plants and diets containing sufficient amounts of antioxidants and essential vitamins are very important factors in treating reproduction and male infertility worldwide. Thus, the aim of this study was to investigate the potential effects of *Kaempferia parviflora (*KP) on the role of some microRNAs in treated and nontreated infertile rats. In addition, the correlation of expressed microRNAs with sperm count, sperm motility, and sperm viability was identified. The probable use of these microRNAs as a diagnostic marker for predicting the clinical response of infertility to the treatment with KP was also achieved.

**Methods:**

In the present study, the potential effects of *Kaempferia parviflora (KP)* at different doses (140, 280, and 420 mg/kg) for six weeks on male rats with subinfertility were explored. In addition, the effect of KP on the expression of circulating microRNAs and its correlation with the parameters of sexual infertility was identified by performing both *in vitro* and *in vivo* assays. *In vitro* antioxidant activity, sperm functional analysis, serum testosterone, and expression of circulating microRNAs were conducted using colorimetric, ELISA, and real-time RT-PCR analysis, respectively.

**Results:**

*Kaempferia parviflora* (KP) at nontoxic doses of 140–420 mg/kg/day for six weeks significantly improved serum testosterone and epididymal sperm parameters (sperm count, motility, and sperm viability), increased testicular weight, and provided a reduction in the percentage of abnormal spermatozoon in infertile male rats. The expression of miR-328 and miR-19b significantly decreased, and miR-34 significantly increased in infertile rats treated with KP compared to infertile nontreated rats. After six weeks of KP therapy, the change in the expression levels of miRNAs was correlated positively with higher levels of serum testosterone and the measures of epididymal sperm parameters. The respective area under the receiver operating characteristic curve (AUC-ROC) was applied to predict the potential use of miR-328, miR-19b, and miR-34 in the diagnosis of male infertility in treated and nontreated infertile male rats. The data showed that AUC cutoff values of 0.91 for miR-328, 0.89 for miR-19b, and 0.86 for miR34 were the best estimated values for the clinical diagnosis of male rats with infertility. In rats treated with KP for six weeks, AUC cutoff values of 0.76 for miR-328, 0.79 for miR-19b, and 0.81 for miR-34 were the best cutoff values reported for the clinical response of infertility to KP therapy after six weeks.

**Conclusions:**

In this study, the improvement of male infertility might proceed via antioxidant and antiapoptotic pathways, which significantly improve spermatogenesis and aphrodisiac properties of males. In addition, the expression of miRNAs, miR-328, miR-34, and miR-19b, in KP-treated and nontreated infertile rats significantly correlated with increased serum testosterone levels and epididymal sperm parameters as well. MicroRNAs, miR-328, miR-34, and miR-19b, might be related to oxidative and apoptotic pathways that proceeded in spermatogenesis. Thus, the use of miRNAs could have a role as diagnostic, therapeutic, and predictive markers for assessing the clinical response of *Kaempferia parviflora* treatment for six weeks. This may have potential applications in the therapeutic strategies based on herbal plants for male infertility. However, in subsequent studies, the genetic regulatory mechanisms of the expressed miRNAs should be fully characterized.

## 1. Introduction

In life, infertility is considered one of the most health problems facing 30–50% of males world wide [1, 2]. Previously, defects in male spermatogenesis, reduction in sperm quality, and seminal production were greatly affected by several treating conditions such as hypogonadism, varicocele, infections, and obstructions [1–3]. In addition, spermatogenesis and sperm normal production were shown to be affected by inadequate vitamins intake, chemotherapy, type of drugs used, toxins, and polluted air [3]. Diets containing sufficient amounts of antioxidants and vitamins A, B, C, and E can enhance barrier stability of testis by increasing blood flow and protect sperm DNA from cellular oxidative-free radical activity [4, 5]. Antioxidants were shown to protect DNA and other cellular components from oxidation and damage, improving sperm quality, which in turn raises the rates of fertility among males [6–8].

Therefore, therapeutic strategies based on herbal plants are very important factors in treating reproduction and male infertility. Natural plants are concomitantly used as a possibility traditional medicine for treating male infertility and other human diseases in up to 60% of the world's population [9–12]. Medicinal plants related to the family Zingiberaceae are used worldwide as spices and are shown to have versatile medical activities, particularly as antioxidative [13], free radical scavenging activities [14, 15], androgenic activity [16], aphrodisiac [17, 18], anticancer [19], and anti-inflammatory [20].

*Kaempferia parviflora* (KP) is one of the most popular plants in the family Zingiberaceae. It has many active constituents like 7-dimethoxyflavone and 5,7,40-trimethoxyflavone [17–24]. Traditionally, KP and its compounds are used as a folk medicine for managing a variety of diseases, particularly male infertility, due to its aphrodisiac, antioxidant, and anti-inflammatory activities [17–22].

Previously, a number of biological activities of KP were identified, particularly antioxidant, anti-inflammatory, and inhibition of NO production, increasing male libido and erectile dysfunction, having aphrodisiac properties, and being used to improve sexual activities and performance [24–28].

In rabbit semen, KP (Krachaidum, KD) showed previously a quiet tendency to increase ejaculation volume and a subsequent increase of the total number, viability, and progressive motility of spermatozoa [29]. Additionally, the seminal vesicle and spermatogenesis significantly improved in rats, following the use of tea or extracts from KP (Krachaidum, KD) [30].

This might be due to the presence of active components like phenolics and flavonoids present mainly in the KP extracts [24]. In other studies, like other plants (curcuma and ginger) in the Zingiberaceae family, it was reported that KP extracts modulate changes in reproductive function by relaxation of the smooth muscles of the blood vessels [31–33], leading to an increase in blood flow to the reproductive organs and finally an improvement in functions of male reproductive organs. Also, KP in association with physical exercise interventions significantly stimulated both increase in sexual motivation and enhancement of sexual performance as well [34].

However, little is known about the roles of circulating miRNAs in reproductive function and male infertility in cases treated with traditional medicine particularly, KP extracts. It was reported previously in many studies that microRNAs as short noncoding transcripts of up to 22 nucleotides have considerable potential as diagnostic and therapeutic tools against many diseases [35, 36]. At the posttranscriptional level, microRNAs might regulate gene expression. So, it could be used for monitoring diagnosis and for treatment of male infertility with therapeutic or herbal medicine [35–37]. In addition, a set of miRNAs was shown to regulate significantly more biological processes like embryonic development, cell differentiation, cell cycle, cell growth, and apoptosis [38–40]. Thus, dysregulation of miRNA functions can lead to the development of disease. miRNAs are shown to contribute to human spermatogenesis and to be retained after the completion of spermatogenesis, and any changes in the expression of spermatozoal RNAs have been associated with male infertility [41–44].

The role of small noncoding RNAs was significantly reported in male germ cell development [45, 46]. In previous studies, miRNAs were reported to have a role in male and female gametogenesis and the development of the embryo [46, 47]. miRNAs were identified in the male reproductive system and in testis, epididymis, sperm cells, seminal plasma, and extracellular vesicles (i.e., exosomes and microvesicles) were suggested to represent known functions. Thus, any alterations in spermatogenesis and embryogenesis could be attributed to the change in the expression of miRNAs [48–54]. These signs could clearly have the potential association of miRNAs in various forms of infertility [53, 54]. In the testis, the critical role of miRNAs was demonstrated during mitotic proliferation and formation of spermatogonia from germ cells. Additionally, their roles also start during spermatogonial stem cells (SSCs) in the epithelium of seminiferous tubules or during spermatocyte meiosis and spermiogenesis [55].

In normozoospermic controls and in infertile males, miR-19b and other miRNAs were clearly expressed in human seminal plasma from fertile controls; however, they significantly increased in the seminal plasma of the infertile men [56]. Thus, a significant increase in the expression levels of miR-19b may be a possible indicator of the degrees of spermatogenic failure in treated and nontreated cases. In addition, other studies reported the expression of many miRNAs, including miR-34, which were associated with many vital processes of male fertility, particularly the regulation of germ cell function as well as cell differentiation during spermatogenesis [56, 57]. It was reported that lower expression and hypermethylation of the promotor of cellular miR-34 types were significantly identified in infertile males. Thus, it was reported that obvious lower expression with hypermethylation of the promoter region makes miR-34 types be an indicator of the deficiency of spermatogenesis [58].

In animal models, inactivation and lower expression of miR34-b,c along with others miRNAs clusters caused low sperm counts, abnormal sperm morphology with low motility, and subsequent male infertility [57, 59, 60]. This might be due to unsuitable or hypermethylation of CpG in their promoter regions. In *n* somatic cells, miR-34 types additionally act as tumor suppressor genes aside from the P53 gene [61].

Also, an increase in the expression levels of miR-328 was reported to govern the pathogenesis of male erectile dysfunction (ED) in many ways. It was found that miR-328 might impair stem cell or neuronal survival, control zonation morphogenesis, and affect calcium homeostasis [62–66].

For aforementioned facts [45–67], identifying the vital role of miR-19b, miR-328, and miR-34 enforces studying their expression profile in treated and nontreated infertility male rats with conventional KP herbal medicine. In addition, there are no scientific reports on the effect of therapeutic or herbal-based treatments such as KP on the role of these circulating microRNAs in reproductive function and male infertility. Therefore, the aim of this study was to investigate the potential effects of KP on the role of microRNAs, miR-19b, miR-328, and miR-34, in treated and nontreated infertile rats and also the correlation of expressed microRNAs with sperm count, sperm motility, and sperm viability, as well as its potential use as diagnostic biomarkers in predicting the clinical response of *Kaempferia parviflora* treatment.

## 2. Materials and Methods

### 2.1. Plant Material

The *Kaempferia parviflora* (KP) rhizomes obtained were purchased from a convenience store (Othaim Markets) in Riyadh, KSA. The plant rhizomes were cut into small pieces and dried in a hot air oven at 55°C [28, 65]. Then, the dried materials were macerated in ethanol twice, for 3 days each, and filtered. To prepare a 1% of fresh KP suspension, the dry KP powder was suspended in distilled water with Tween 80 [28, 65].

### 2.2. Assessment of Total Phenolic Content (TPC) and Total Flavonoid (TF) Content

#### 2.2.1. Preparation of KP Extract

In this test, a mechanical blender was used to prepare a fine powder of the dried rhizomes of KP. At room temperature, the rhizomes of the plant were dried in the shade and then chopped into small pieces. KP was ground to a fine powder and became ready for the extraction step. By using a Soxhlet apparatus, 20 g of the dried rhizome powder was extracted in 300 mL methanol at 60–65°C for 3-4 h. Then, Whatman filter paper No. 1 was used to filtrate the extract and exposed to pressure at 40°C for the concentration process. Finally, the extract was further dried, weighed (2.6 g), and stored in storage vials at 4°C for reuse in the study [28, 65, 66].

#### 2.2.2. Total Phenolic Content

In this experiment, the total phenolic content of the KP extract was estimated by using Folin–Ciocalteu method as previously reported [68, 69]. “A total of 200 *μ*L of crude KP extract (1 mg/mL/3 mL dH_2_O) was mixed thoroughly with 0.5 mL of Folin–Ciocalteu reagent for 3 min. To the mixture, 2 mL of 20% (w/v) sodium carbonate was added, and the whole mixture was stored in the dark for 60 min as mentioned previously” [68, 69]. Then, “the absorbance of the produced mixture was measured at 650 nm. Finally, calibration curves were used to calculate the concentrations of the total phenolic contents and expressed as mg of gallic acid equivalent per g dry weight as mentioned before” [68, 69].

#### 2.2.3. Total Flavonoid Content

In this test, the aluminum chloride colorimetric method was used to estimate the total flavonoid content of crude KP extract, as mentioned previously [70]. In this experiment, “50 *μ*L of KP extract (1 mg/mL ethanol) was completed to 1 mL with methanol and mixed with 4 mL of dH_2_O. Moreover, after 5 min of incubation, 0.3 mL of 5% NaNO_2_ solution and 0.3 mL of 10% AlCl_3_ solution were added, and the mixture was allowed to stand for 6 min [70], followed by the addition of 2 mL of 1 mol/L NaOH solution and the final volume of the mixture reached to 10 mL by adding dH_2_O” [70]. “The absorbance of the mixture was measured after 15 min. Finally, from a calibration curve, the concentration of the total flavonoid content present was calculated, and the result was expressed as mg rutin equivalent per g dry weight” [70].

### 2.3. Assessment of Antioxidant Activity

#### 2.3.1. DPPH Assay

The 1,1-diphenyl-2-picryl-hydrazyl (DPPH) was used to estimate the antioxidant activity of the KP extract as mentioned before [71]. “In this test, a mixture of the KP extract was prepared, whereas 3.8 mL DPPH solution was added to 200 *μ*L of each extract (100–500 *μ*g/mL) and the whole mixture left in the dark for one hour at room temperature as mentioned before. In the final stage, the KP mixture was subjected to measure the absorbance at 517 nm against ascorbic acid as a positive control” [71]. Finally, “the ability of the sample to scavenge DPPH radical was determined as follows” [71]:

  {DPPH scavenging effect = Control OD − Sample OD/Control OD × 100}---[71].

#### 2.3.2. Nitroblue Tetrazolium (NBT) Assay

The free radical scavenging activity of KP extract to superoxide anion was identified by nitroblue tetrazolium (NBT) as previously reported [72]. “In this test, a total of 100–500 *μ*g/mL of the KP extract reacted with a mixture of 1.5 mmol/L riboflavin, 50 mmol/L nitroblue tetrazolium (NBT), 10 mmol/L D,l-methionine, and 0.025% (v/v) Triton X-100 in 50 mmol/L phosphate buffer at pH 7.8, respectively” [72]. “Then, the reaction mixture was initiated by the illuminating process to produce a colored formazan compound. The absorbance of formazan was recorded at 560 nm against ascorbic acid as a positive control. Finally, the percentage of the scavenging activity was identified as the inverse of the produced formazan” [72].

#### 2.3.3. FRAP Assay

In this experiment, the antioxidant capacity of the KP extract was identified by estimation of ferric (Fe +3) reducing antioxidant power (FRAP), as mentioned previously [73]. “In this method, 100 *μ*L of the KP extract (100–500 *μ*g/mL) was incubated with 2.5 mL of 200 mmol/L phosphate buffer (pH 6.6) and 2.5 mL of1% potassium ferricyanide for 20 min at 50°C. After that, 2.5 mL of 10% trichloroacetic acid was added to the reaction mixture and was centrifuged at 10,000 rpm for10 min” [73]. “Then, a mixture from the upper layer was performed, whereas 5 mL of this layer was mixed with 5.0 mL dH_2_O and 1 mL of 0.1% Fe Cl_3_. The reaction mixtures were subjected to measure the absorbance at 700 nm against ascorbic acid as a positive control” [73].

### 2.4. Acute Toxicity Test

In this test, although different concentrations of KP (140, 280, and 420 mg/rat) were previously studied as improving agents for sexual performance in streptozotocin- (STZ-) induced diabetic male rats with infertility [28], the cytotoxicity of KP extract at doses of 140, 280, and 420 mg/rat was subjected to measure cellular toxicity in a healthy group of rats (10 rats) as previously reported in many toxicity studies [74, 75]. After the first 4 h of dosing, all animals have been observed for the appearance of any symptoms of toxicity. In addition, the survived animals were recorded following 24 h and maintained under daily observations for two weeks [74–76].

### 2.5. Animals Care and Experimental Design

Fifty adult male Wistar rats weighing about 180–200 g were included in this study. One week before starting the experiment, all rats were allowed to acclimatize to the laboratory environment like controlled conditions of the light cycle (12 hr : 12 hr, light : dark), room temperature (25 ± 2°C), and relative humidity (60%–70%) with free access water and rat chow. Animals were randomly classified into five groups of 10 animals each. Group 1 (normal group) was administered with the vehicle (distilled water), and group 2 (subinfertility group) received para-amino salicylic acid (PAS) at a dose of 400 mg/kg bw, while groups 3, 4, and 5 were given PAS at a dose of 400 mg/kg bw with an aqueous suspension of KP extract at doses of 140, 280, and 420 mg/kg, respectively [28, 75]. The animals were orally administered KP once a day for 6 weeks. “The experiment and the experimental procedures were performed according to the guidelines of the Experimental Animal Care Center, College of Applied Medical Sciences, and were approved by the Ethics Committee of the Experimental Animal Care Society, RRC, College of Applied Medical Sciences, King Saud Univ., Riyadh, Saudi Arabia, under file number (RRC-2019-021)” [76].

### 2.6. Sperm Collection and Functional Analysis

An overdose of “pentobarbital sodium was applied for anesthesia; then, all animals were sacrificed to collect the testes and epididymis” [28]. “Before the collection of spermatozoa, the testes and epididymis were weighted. In addition, to collect rat spermatozoa, a cauda part of the epididymis was minced into small pieces and mixed in 1 ml of Hanks' balanced salt solution prewarmed at 37°C. Also, sperm parameters such as sperm count, motility, and viability were examined by microscope as previously mentioned” [77].

A Neubauer cell counting chamber under 10× magnification was used to collect sperm counts, as mentioned previously [78]. In addition, “the one-step eosin-nigrosin staining technique was applied to assess the percentage of sperm viability and morphology like normality and abnormality” [77, 78]. “In this test, sperm viability and morphology were then evaluated by counting alive and dead cells, whereas nonstained cells were considered alive and orange-red colored cells were considered dead cells” [77].

### 2.7. Assessment of Serum Testosterone

Serum samples were collected from the blood by “centrifugation at 2200 g for 15 min at 4°C and subjected to testosterone analysis using immunoassay ELISA Kit (Testosterone ELISA Kit, Abcam, Cambridge, UK)” [28, 65]. The level of testosterone in each sample was calculated according to the manufacturer's instructions, as mentioned before [28, 65].

### 2.8. Real-Time RT-PCR Analysis of Circulating miRNAs

#### 2.8.1. Extraction of RNA and Synthesis of *cDNA*

In this experiment, “RNA of all samples was estimated by using a reverse transcription-polymerase chain reaction (RT-PCR) analyses and the miRNease isolation kit (Qiagen, Hilden, Germany) as mentioned previously” [79–82]. Then, “reverse transcription miScriptII RT kits (Qiagen) were applied to generate a complementary DNA (cDNA), and then the levels of miRNAs were evaluated by optical density” [79–82].

#### 2.8.2. Real-Time RT-PCR Analysis

The expression of “miRNAs in the serum was identified by using quantitative real-time RT-PCR analyses and primers of circulating miRNAs, miR-328, miR-34, and miR-19b (Applied Biosystems, Foster City, CA, USA)” [79]. In this test, “GAPDH gene was applied as an internal housekeeping gene to normalize the average copy number of the resultant PCR components as previously stated in the literature” [80–82].

In PCR process, “templets of respective cDNA were subjected to four thermal phases: primary denaturation phase (I) (at 94°C for 2 minutes); denaturation phase (II) (at 94°C for 30 seconds); annealing phase (III) (at 59°C for 30 seconds); and amplification phase (IV) (at 72°C for 30 seconds)” [80–82]. “The PCR phases (II to IV) proceeded for 45 cycles, and all reactions were measured in a triplicate manner” [80–82].

#### 2.8.3. Statistical Analysis

In this study, “the data obtained were analyzed by using an SPSS statistical program (SPSS, IBM Statistics V.17) and the results of the continuous variables were expressed as mean ± SD” [82]. In addition, the “nonparametric test (Mann–Whitney-Wilcoxon test) and the *χ*2 test were performed to estimate the frequency differences between the groups, respectively” [82]. Moreover, “to compare between the studied variables like serum testosterone levels, sperm viability and morphology, and expression levels of miRNAs, two independent sample *t*-tests were used for all groups. Additionally, multiple stepwise regressions and Pearson's correlations analysis were used to estimate the association between the expressed miRNAs and the studied independent variables in KP-treated and nontreated rats” [82]. “The area under the receiver operating characteristic (ROC) curve was used to measure the susceptibility and sensitivity of the studied parameters like testosterone, sperm viability, morphology, and miRNAs, miR-328, miR-34, and miR-19b, for the diagnosis of male infertility in treated and nontreated rats as previously reported” [82]. All tests were two-tailed; because of multiple assessments, results were only considered statistically significant at a value of *p* < 0.05.

## 3. Results

### 3.1. Phenolic and Flavonoid Contents

Total phenolic and flavonoids constituents were calculated from the calibration curves at *R*^2^ = 0.965 for total phenolic content and *R*^2^ = 0.986 for the total flavonoid content, respectively. The total phenolic content estimated from the methanolic KP extract was 76.8 ± 3.8 gallic acid equivalents/g, and the total flavonoid content was 42.8 ± 2.7 rutin equivalents/g (Table 1).

### 3.2. Antioxidant Activity

**Figure 1:**
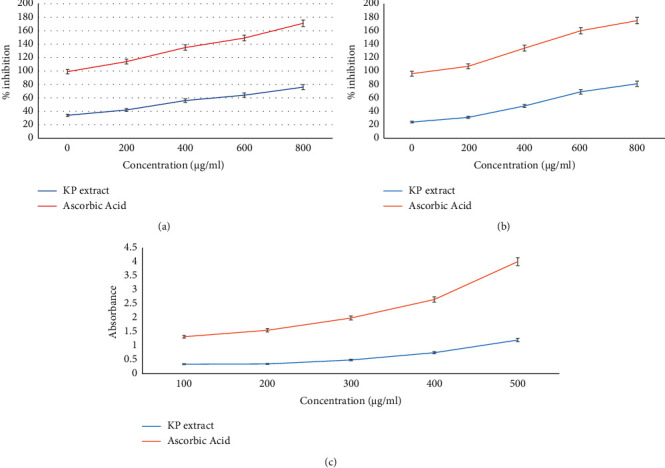
Antioxidant and free radical scavenging activities of the *Kaempferia parviflora Rhizome.* (a) Free radical scavenging activity, (b) superoxide scavenging activity, and (c) ferrous reducing capacity of methanolic extracts of the *Kaempferia parviflora Rhizome.* Ascorbic acid was included as a positive control. Each value is the mean ± standard deviation.

The biological antioxidant activity of *Kaempferia parviflora* (KP) was measured *in vitro* and calculated against the activity to scavenge DPPH radical, nitroblue tetrazolium (NBT), and ferric (Fe +3) reducing antioxidant power (FRAP) as shown in Figure 1. The methanolic rhizome extract of *Kaempferia parviflora* had strong antioxidant activity against all the free radicals investigated. The DPPH radical is widely used in assessing free radical scavenging activity because of the ease of the reaction. DPPH scavenging activity was 67.13% at a concentration of 600 *μ*g/mL rhizome extract, while that of the control, ascorbic acid, was 85% (Figure 1(a)). The data showed that the KP extract scavenging activity against DPPH radicals is a concentration-dependent manner that significantly increased with higher concentrations (Figure 1(a)). Also, in the NBT assay, superoxide scavenging activity determined for KP extract was 69.0% for 600 *μ*g/mL of the rhizome extract and 91.3% for ascorbic acid (Figure 1(b)). The superoxide scavenging activity is also increased in a dependent manner with higher concentrations (Figure 1(b)).

In assays of the reducing power of the crude extract, significant changes in absorbance at 700 nm were observed (0.34–1.2) with increasing concentrations of extract (100–500 *μ*g/mL) compared to that calculated for ascorbic acid (0.98–2.8) at the same respective concentrations of extract (100–500 *μ*g/mL) as shown in Figure 1(c).

### 3.3. Acute Toxicity Test

In the acute toxicity test, the rats showed no toxicity and lethality (LD50 value = 0) following administration of various doses of KP extract, 140, 280, and 420 mg/rat. The results showed that KP extracts have no toxicity event at a higher dose of KP 420 mg/kg.

### 3.4. Effect of KP Extract on Testis, Epididymis, and Seminal Vesicle Weight

As shown in Table 2, the inductions of male infertility cause a significant decrease in the weights of testes, epididymis, and seminal vesicle compared to normal rats (*p* < 0.05). On the other hand, treatment of infertile rats with KP at different doses (140 mg/kg, 280 mg/kg, and 420 mg/kg) significantly (*p* < 0.01) improved testicular weight when compared to nontreated subinfertile rats. A high dose of KP (420 mg/kg) improved the epididymis weight and seminal vesicle compared to nontreated rats (*p* < 0.001).

### 3.5. Effect of KP Treatment on Sperm Parameters and Functional Analysis

In this experiment, sperm count, sperm motility, and sperm viability have been significantly reduced, and morphological abnormality in sperms has increased in the infertile rats compared to (*p* < 0.05) normal group, as shown in Table 3. Compared to nontreated infertile rats, a significant increase (*p* < 0.01) in sperm count, sperm motility, and sperm viability and a reduction in the percentage of abnormal spermatozoon were reported in infertile rats following treatment with KP at doses of 140 mg/kg, 280 mg/kg, and 420 mg/kg, respectively (Table 3). Sperm parameters significantly increased and abnormal spermatozoon significantly improved in infertile rats treated with KP at a dose of 420 mg/kg compared (*p* < 0.001) to the respective KP doses, which signifies the activity of KP extract via a dependent manner with higher concentrations (Table 3).

### 3.6. Effect of KP Treatment on Serum Testosterone Concentration

**Figure 2:**
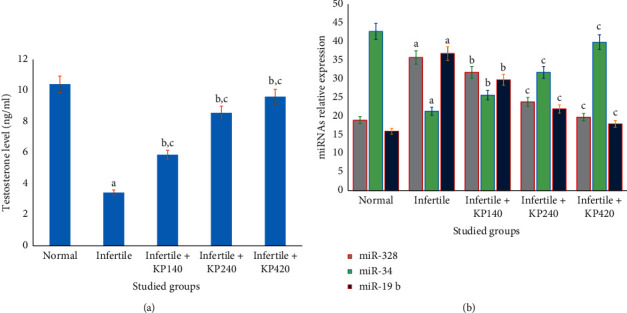
Effect of the *Kaempferia parviflora Rhizome* on the expression of serum testosterone and microRNAs' differential profile in normal, treated, and treated infertile rats. The results showed that serum testosterone levels significantly increased in rats treated with KP extracts at doses of 140 up to 420 mg/kg/day compared to infertile nontreated rats, respectively (a). In addition, the relative expression of miR-328 and miR-19b significantly decreased, and miR-34 significantly increased in infertile rats treated with KP extract at doses of 140 up to 420 mg/kg/day compared to infertile nontreated rats, respectively (b). The improvement in the levels of serum testosterone and the differential expression of microRNAs is in a dose-dependent manner. ^a^*p* ≤ 0.05, ^b^*p* ≤ 0.01, and ^c^*p* ≤ 0.001.

The results showed that serum testosterone levels were significantly (*p* < 0.05) reduced in infertile rats compared to normal controls (Figure 2(a)). Serum testosterone levels significantly (*p* < 0.001) increased in infertile rats treated with KP extracts at doses of 140 up to 420 mg/kg/day compared to infertile nontreated rats, respectively (Figure 2(a)).

### 3.7. Effect of KP Treatment on MicroRNAs' Differential Expression Profile

In this experiment, the relative expression of cellular miRNAs was significantly identified by quantitative RT-PCR analysis in control, KP-treated, and nontreated infertile rats, as shown in Figure (2(b)). In infertile rats, the expression levels of both miR-328 and miR-19b significantly increased, and miR34 significantly reduced compared (*p* < 0.05) to healthy normal rats, as shown in Figure 2(b). The treatment of infertile rats with KP extract at doses of 140 up to 420 mg/kg/day significantly improved the expression levels of cellular miRNAs, whereas the relative expression of miR-328 and miR-19b significantly decreased, and miR-34 significantly increased in infertile rats compared to (*p* < 0.01) infertile nontreated rats, respectively (Figure 2(b)). The improvement in the differential expression of microRNAs is a dose-dependent manner, whereas it is significantly more improved at a higher KP dose of 420 mg/kg/day compared to (*p* < 0.001) respective lower KP doses (Figure 2(b)). Moreover, the cellular expression of miR-328, miR-19b, and miR34 significantly correlated with the improved epididymal sperm parameters (sperm count, motility, viability, and abnormal morphology in infertile rats treated with different doses of KP extract for sex weeks as shown in Table 4, signifying that the expression of miR-328, miR-19b, and miR34 in serum was correlated with male infertility.

To define the possible use of miR-328, miR-34, and miR-19b expression levels as diagnostic biomarkers of male infertility, ROC analysis was performed (Table 5). The data showed that the AUC was 0.91 (0.88–0.96) for miRNA-328, with a sensitivity of 85.6% and specificity of 89.5%, for miRNA-34 AUC was 0.86 (0.78–0.91), with a sensitivity of 89.3% and specificity of 91.3%, and for miR-19b AUC was 0.89 (0.81–0.98), with a sensitivity of 79.5% and specificity of 82.5% at the best cutoff values as shown in Table 5, which indicates that the miR-328, miR-34, and miR-19b levels were strong cellular molecular predictors for diagnosis and treatment of male infertility.

**Figure 3:**
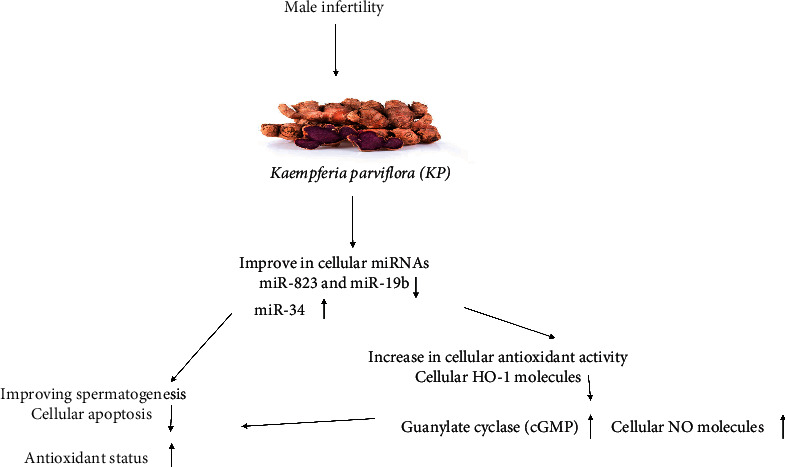
Proposed role of the effect of the *Kaempferia parviflora Rhizome* on male subinfertility.

In addition, baseline expression of miR-328, miR-34, and miR-19b was analyzed for the treatment response to KP extract at 6 weeks using the ROC curve. It was noticed that the AUC was 0.76 (0.65–0.86) for baseline miRNA-328 for clinical response at 6 weeks, with a sensitivity of 76.8% and a specificity of 79.7%, the AUC was 0.81 (0.78–0.96) for baseline expression of miRNA-34, with a sensitivity of 69.8% and a specificity of 71.8%, and AUC was 0.79 (0.65–0.88) for miR-19b, with a sensitivity of 81.2% and specificity of 79.3%, respectively, at the best cutoff values (Table 5). These data recognized that miR-328, miR-34, and miR-19b expression in serum might have values in predicting the clinical response of *Kaempferia parviflora* treatment. In this study, the proposed role of the *Kaempferia parviflora Rhizome* on male subinfertility proceeded via improving the expression of miRNAs, miR-328, miR-34, and miR-19b, which in turn improves the spermatogenesis through antioxidant and antiapoptosis (Figure 3).

## 4. Discussion

In this study, the results suggested that daily administration of *Kaempferia parviflora* (KP) at doses of 140 up to 420 mg/kg has a beneficial effect on male reproductive functions in infertile male rats. Our data showed that testosterone, sperm count, motility, and sperm viability significantly increased, and the percentage of abnormal spermatozoon significantly reduced in treated infertile rats. In addition, the absolute testicular and epididymis weights were significantly increased in treated rats, which might be related to increased testosterone.

In this study, the applied doses of KP extracts against male infertility showed no acute or chronic toxicity. The results displayed no cellular toxicity and lethality (LD50 value = 0) observed up to 420 mg/kg of KP extract in the animals. Like other studies, there are no abnormal changes in body weight and histology in various visceral organs following oral administration of KP extracts [83, 84]. At the tested doses, no negative effects on renal and hepatic functions were reported. Previously, toxicological studies showed no changes in hemoglobin, white blood cells, or differential cell count following the administration of ethanolic KP extracts at the doses of 60, 120, and 240 mg/kg for 60 days, respectively [85, 86].

Previously, it was reported that secondary metabolites present in plants like phenolics, flavonoids, and carotenoids are rich in antioxidant activity, which might be due to their redox properties and chemical structures [87–90]. Thus, enhancement in the reproductive function of male rats following administration of KP extracts might be due to the antioxidant, reducing, and free radical scavenging activities of their total phenolic and flavonoids constituents [91].

In this study, considerable amounts of total phenolic (76.8 ± 3.8) and flavonoids (42.8 ± 2.7) were identified in the methanolic KP extract. Phenolic and flavonoid compounds present in all plants have redox properties, which allow them to behave as active as antioxidants [86]. The presence of active hydroxyl groups in phenolic and flavonoids compounds is related to their free radical scavenging abilities *in vitro* and in *in vivo* processes [92–95].

In addition, the *in vitro* antioxidant activity of KP extract was identified in this study. The methanolic rhizome extract of *Kaempferia parviflora* (KP) had strong antioxidant activity against all investigated free radicals. The data showed that scavenging activity DPPH and superoxides were 67.13% and 69.0%, respectively. In addition, the reducing power of the crude KP extract measured by FRAP was observed at 0.34–1.2, respectively, with increasing concentrations of extract (100–500 *μ*g/mL) at 700 nm. It was reported that cellular superoxide-free radicals initiated within the cells by external initiators significantly lead to cellular destruction and DNA damage [84–88]. Like other plants, KP extract is enriched with higher amounts of both phenolic and flavonoids, which are responsible for their bioactivity against cellular oxidative-free radicals implicated in several diseases [89]. In addition, flavonoids like phenols were shown to suppress reactive oxygen formation, chelated trace elements involved in free radical production. It acts to scavenge reactive species and upregulate and protect antioxidant defenses [84–90].

Thus, the potential effect of antioxidant and free radical scavenging activity of KP extract on male rat infertility was identified. Serum levels of testosterone, sperm count, sperm motility, and sperm viability significantly increased and the ratios of abnormal forms of sperms significantly reduced in infertile rats treated with different doses of KP extract (140–420 mg/kg) compared to nontreated infertile rats, respectively. The improvement in fertility parameters increased in a dose-dependent manner, whereas higher KP doses (420 mg/kg) showed the greatest improvement in serum testosterone along with improved sperm quality.

Like our results, KP treatment was reported previously to elevate serum testosterone which in turn increases the weight of the reproductive organ [26, 90]. In infertile rats treated with KP, the increase in the level of serum testosterone is significantly associated with the development of male reproductive organs and an increase in sexual activity [91]. Also, KP treatment in infertile male rats enhances sexual motivation [90], sexual behavior [24], and sexual performance via elevating blood flow to the testis, enhancing NO production in the medial preoptic area (MPOA) [90].

In infertile rats treated with KP extracts, an improvement in the aphrodisiac properties was reported. The aphrodisiac properties include enhanced serum testosterone levels, increased testicular weight, increased sperm density, and improved sexual performance.

Previous studies confirmed that KP phytoconstituents like 5,7-dimethoxyflavone and 5,7,40-trimethoxyflavone were reported to have inhibitory activity against phosphodiesterase 5 (PDE5), suppression of NO production, increasing male libido, and improving erectile dysfunction in treated males [17, 18, 23–25, 90]. Also, these polyphenols possess aphrodisiac properties which are being used to improve sexual activities and performance [24–28].

To explore the role of some molecular circulating microRNAs in diagnosis and clinical response to therapeutic strategies for treating male infertility, quantitative RT-PCR analyses were performed to evaluate the expression of miR-328, miR-34, and miR-19b as diagnostic biomarkers of male infertility.

In infertile male rats treated with KP extract at doses of 140 up to 420 mg/kg/day, there were a significant increase in the expression level of miR-34 and a reduction in the expression levels of both miR-328 and miR-19b, respectively, compared to nontreated rats. The change in the expression of the studied miRNAs significantly improved at applied doses of KP extracts which confirms the effect of KP on cellular infertility via dose-dependent manner, particularly spermatogenesis. The proposed role of miR-328, miR-34, and miR-19b was previously proposed as promotors or modulators of spermatogenesis [41–67]. Previously, miRNAs were classified as imperative cellular regulators of various biological processes, including spermatogenesis [91]. The aberrant expression of miRNAs was shown to affect spermatogenesis at multiple stages and in different cell types, and most of them often resulted in infertility [91–95]. In addition, it was reported that miRNAs like miR-1, miR-200a, miR-203, and miR-206 might play a role in the development of erectile dysfunction (ED) in rats. They can regulate the endothelial nitric oxide (NO) synthase, NO, protein kinase G, the prostaglandin E1, protein kinase A pathways, and the development of ED [96].

In rats with infertility, upregulation of miR-328a was shown to be associated with the onset of male erectile dysfunction (ED), whereas miR-328a might be required for the downregulation of the molecule, that is, HO-1, the most vital molecule in mediating erectile function [42]. In addition, in rats, cellular HO-1 molecules generate carbon monoxide, which positively affects the levels of guanylate cyclase and cGMP present in the vascular endothelial cells and cavernous tissue, respectively [42].

Moreover, HO-1 molecules play a role in the improvement of cellular antioxidants via scavenging reactive oxygen species and prevent NO from reacting with reactive oxygen species and forming peroxynitrite [45]. Thus, in this study, downregulation of miR-328a following the treatment with KP extracts reduces HO-1 gene expression and reduces cellular oxidative stress and release of more NO, which finally might enhance erectile function and produce more male fertility [45]. In addition, increased levels of miR-328 imply that it might govern male infertility and ED by affecting calcium homeostasis, as previously reported [29, 38, 97]. Moreover, miRNAs were studied in the testicular tissue, seminal plasma, or spermatozoa that also might be associated with male subfertility [66, 98–100].

Several studies have revealed that biological fluids such as semen, blood, saliva, vaginal secretions, and menstrual blood have specific miRNAs [54, 101]. Recent studies showed that overexpression levels of miR-19b aberrantly may be an indicator of spermatogenic failure, particularly in idiopathic infertile males. miR-19b was shown to be responsible for cellular apoptosis and expressed at higher levels throughout the development of primordial germ cells (PGCs) and spermatogonia. Thus, it was proposed that miR-19b is very important for the survival and proliferation of spermatogonia [102]. These studies suggest that miR-19b could play a role in regulating spermatogenesis in human males.

In patients with oligozoospermia and nonobstructive azoospermia (NOA), altered miRNA expression in the testis was shown to be associated with infertile testis [103]. miRNAs could be involved in the translational repression of meiotic synapsis during spermatogenesis. Also, the roles miRNAs played in germ cell proliferation and differentiation were identified, which in turn suggest their association with male infertility [104–107].

Expression of miR-34 with other sets of miRNAs was reported in patients with different forms of spermatogenic impairments and compared with their values in normal cases. The data obtained suggested the potential use of miR-34 along with others as novel noninvasive biomarkers to diagnose patients with fertility, subfertility, infertility [108].

In this study, the expression of the targeted miRNAs, miR-328, miR-19b, and miR34, correlated positively with the identified epididymal sperm parameters like sperm count, motility, viability, abnormal morphology, and increased levels of testosterone of treated male rats with KP extract for six weeks, suggesting activation of male aphrodisiac properties via antioxidant, anti-inflammatory, and regulation of cellular NO production. This, in turn, improves male libido and erectile dysfunction and subsequent male fertility and performance [24–28].

Interestingly, different patterns of human testicular histopathology like Sertoli cell only, mixed atrophy, and germ cell arrest were significantly associated with the change in the expression profile of cellular miRNA [109–113]. In addition, both types of spermatogenic impairments and azoospermia of patients were also associated with cellular miRNA, as mentioned previously [109–113].

The use of the expression of miRNAs, miR-328, miR19b, and miR-34, as specified markers for male fertility and clinical therapeutic response to KP treatments was predicted using the analysis of the respective area under the receiver operating characteristic curve (AUC-ROC).

In this study, AUC cutoff values of 0.91 for miR-328, 0.89 for miR-19b, and 0.86 for miR34 were the best assessed for clinical diagnosis of males with infertility, and the AUC cutoff values of 0.76 for miR-328, 0.79 for miR-19b, and 0.81 for miR-34 were the best cutoff values, respectively, reported for the prediction of the clinical response of male infertility following KP therapy for six weeks.

In patients with infertility, abnormal sperm count, motility, and morphology were significantly associated with the expression of miRNAs and increased levels of sperm DNA fragmentation [54, 114, 115]. The rate of fertility significantly reduced with increased DNA fragmentation in spermatozoa [115–118]. The correlation between miRNAs and DNA fragmentation is weak but significant, particularly with higher apoptosis during the spermatogenesis process, especially in spermatogonial and primary spermatocytes compared with that in mature spermatozoa [117–124].

### 4.1. Limitations

In this study, the expression of miR-328, miR-34, and miR-19b in the serum of males with infertility and their correlation with epididymal sperm parameters and hormonal testosterone may have values in predicting the clinical response of *Kaempferia parviflora* treatment. However, target genes that might be responsible for the regulatory mechanisms of miRNAs in male fertility following the application of KP extracts should be fully characterized. Although previous studies reported that KP administration improves sperm parameters, hormonal testosterone, and spermatogenesis via scavenging of cellular oxidative-free radicals and increasing of antioxidants enzymes, additionally targeted genes of oxidative and antioxidant enzymes and their association with miRNAs should be identified. Thus, future deep studies based on genetic analysis of the target genes of miR-328, miR-34, and miR-19b and their relation to the cellular antioxidant activity of KP should be addressed. This might suggest the proposed use of miRNAs in regulating and improving clinical diagnosis of male infertility when used as diagnostic markers in coincidence with the traditional techniques.

## 5. Conclusion

In this study, the expression of miRNAs, miR-328, miR-34, and miR-19b, in KP-treated and nontreated infertile rats was significantly correlated with increased serum testosterone levels and epididymal sperm parameters as well. MicroRNAs, miR-328, miR-34, and miR-19b, could be used as diagnostic, therapeutic, and predictive biomarkers for assessing the clinical response of *Kaempferia parviflora* treatment for six weeks. This may have potential applications in the therapeutic strategies based upon herbal plants for male infertility. However, in subsequent studies, the genetic regulatory mechanisms of the expressed miRNAs should be fully characterized.

## Figures and Tables

**Table 1:** Total phenolics and flavonoids content of methanolic extract of *Kaempferia parviflora Rhizome.*

Phytoconstituents	Quantity
Total phenolics content^a^	76.8 ± 3.8 (*R*^2^ = 0.965)
Total flavonoids content^b^	42.8 ± 2.7(*R*^2^ = 0.986)

Values are means of three biological replicates. ^a^mg gallic acid equivalent (GAE)/g DW. ^b^mg rutin equivalent/g DW.

**Table 2:** Effect of KP extract on testis, epididymis, and seminal vesicle weight.

Groups	Reproductive organ weight (g)		
	Testes	Epididymis	Seminal vesicle
Normal	4.15 ± 0.18	1.82 ± 0.22	1.264 ± 0.25
Subinfertile	2.96 ± 0.47^a^	1.123 ± 0.17^a^	0.76 ± 0.16^a^
Subinfertile + KP140	3.45 ± 0.14^b,c^	1.6 ± 0.12^b,c^	0.98 ± 0.23^b,c^
Subinfertile + KP280	3.65 ± 0.11^b,c^	1.94 ± 0.11^b,c^	1.5 ± 0.13^b,c^
Subinfertile + KP420	3.96 ± 0.18^b,c^	2.36 ± 0.15^b,c^	2.53 ± 0.21^b,c^

Data are shown as the mean ± SD. Significance at *p* < 0.05.^a^*p* < 0.01 (subinfertile versus normal group), ^b^*p* < 0.01 (subinfertile + KP-treated versus subinfertile nontreated or normal group), and ^c^*p* < 0.001 (KP420 groups versus KP140 or KP280 groups).

**Table 3:** Effect of KP extract on epididymal sperm parameters (sperm count, motility, viability, and abnormal morphology) after six weeks of treatment.

Groups	Epididymal sperm parameter			
	Sperm count (million/ml)	Sperm motility (%)	Sperm viability (%)	Sperm abnormality (%)
Normal	14.655 ± 1.5	87.503 ± 5.19	93.5 ± 3.72	3.7 ± 0.86
Subinfertility	8.58 ± 1.21^a^	69.2 ± 4.86^a^	72.7 ± 2.75^a^	5.1 ± 1.75^a^
Subinfertile + KP140	12.70 ± 1.25^b,c^	74.3 ± 3.36^b,c^	76.3 ± 2.45^b,c^	4.6 ± 1.57^b,c^
Subinfertile + KP280	13.40 ± 1.6^b,c^	76.8 ± 3.56^b,c^	84.5 ± 2.8^b,c^	3.86 ± 1.37^b,c^
Subinfertile + KP420	16.80 ± 1.6^b,c^	85.9 ± 4.71^b,c^	91.3 ± 2.71^b,c^	3.7 ± 1.42^b,c^

Data are shown as the mean ± SD. Significance at *p*< 0.05.^a^*p* < 0.01 (infertile versus normal group), ^b^*p* < 0.01 (infertile + KP-treated versus infertile nontreated or normal group), and ^c^*p* < 0.001 (KP420 groups versus KP140 or KP280 groups).

**Table 4:** Correlations between miRNAs expression, serum testosterone levels, and epididymal sperm parameters in KP-treated infertile rats.

Studied parameters	miRNAs expression		
	miR-328	miR-34	miR-19b
Serum testosterone (ng/ml)	0.68^b^	0.54^b^	0.14^b^
Sperm count	0.21^b^	0.75^b^	0.25^b^
Sperm motility	0.19^a^	0.36^b^	0.16^b^
Sperm viability	0.23^a^	0.43^b^	0.18^b^
Sperm abnormality	−0.24^b^	−0.31^b^	−0.22^a^

Data are presented as Pearson's (*R*) coefficients adjusting for variables identified as cofounders in univariate analyses. Significance at *p* < 0.05. ^a^*p* < 0.01 and ^b^*p* < 0.001.

**Table 5:** ROC cutoff values of miR-328, miR-34, and miR-19b for diagnosis and treatment of rat infertility with KP extract (140–420 mg/kg/day) for six weeks (*n* = 50).

miRNAs	AUC for diagnosis of male infertility (a)			AUC for clinical response to KP therapy at six weeks^(b)^		
	Area (95% CI)	Sensitivity	Specificity	Area (95% CI)	Sensitivity	Specificity
miR-328	0.91 (0.88–0.96)	85.6	89.5	0.76 (0.65–0.86)	76.8	79.9
miR-34	0.86 (0.78–0.91)	89.3	91.3	0.81 (0.78–0.96)	69.8	71.8
miR-19b	0.89 (0.81–0.98)	79.5	82.5	0.79 (0.65–0.88)	81.2	79.3

AUC = area under the curve. CI = confidence interval. ^a^*p* < 0.05, ^b^*p* < 0.01, and ^c^*p* < 0.001.

## Data Availability

All the data generated or analyzed during this study are included in the manuscript. Please contact the corresponding author for access to the data presented in this study.

## References

[1] Isidori A. M., Pozza C., Gianfrilli D., Isidori A. (2006). Medical treatment to improve sperm quality. *Reproductive BioMedicine Online*.

[2] Shefi S., Turek P. J. (2006). Definition and current evaluation of subfertile men. *International Brazilian Journal of Urology*.

[3] Carlsen E., Giwercman A., Keiding N., Skakkebaek N. E. (1992). Evidence for decreasing quality of semen during past 50 years. *BMJ*.

[4] Mosher W. D., Pratt W. F. (1991). Fecundity and infertility in the United States: incidence and trends∗∗The views expressed in this editorial are solely those of the authors and not necessarily those of the U.S. Department of Health and Human Services. *Fertility and Sterility*.

[5] Lu P., Lai B. S., Liang P., Chen Z. T., Shun S. Q. (2003). Antioxidation activity and protective effection of ginger oil on DNA damage in vitro. *Zhongguo Zhong Yao Za Zhi*.

[6] Jedlinska-Krakowska M., Bomba G., Jakubowski K., Rotkiewicz T., Jana B., Penkowski A. (2006). Impact of oxidative stress and supplementation with vitamins E and C on testes morphology in rats. *Journal of Reproduction and Development*.

[7] Rajeev K., Gagan G., Narmada P. (2006). Drug therapy for idiopathic male infertility: rationale versus evidence. *Journal of Urology*.

[8] Yang H. S., Han D. K., Kim J. R., Sim J. C. (2006). Effects of *α*-tocopherol on cadmium-induced toxicity in rat testis and spermatogenesis. *Journal of Korean Medical Science*.

[9] Larsen U., Hollos M. (2005). The importance of motherhood: a study of infertility in urban Northern Tanzania.

[10] Feldman R. H., Laura R. (2004). The use of complementary and alternative medicine practices among Australian university students. *Complementary Health Practice Review*.

[11] Rates S. M. K. (2001). Plants as source of drugs. *Toxicon*.

[12] Chapman K. R., Chomchalow N., Batugal P. A., Kanniah J., Lee S. Y., Oliver J. T. (2003). Production of medicinal plants in Asia. *Medicinal Plants Research in Asia, Vol 1. The Framework and Project Workplans*.

[13] Sekiwa Y., Kubota K., Kobayashi A. (2000). Isolation of novel glucosides related to gingerdiol from ginger and their antioxidative activities. *Journal of Agricultural and Food Chemistry*.

[14] Zancan K. C., Marques M. O. M., Petenate A. J., Meireles M. A. A. (2002). Extraction of ginger (Zingiber officinale Roscoe) oleoresin with CO_2_ and co-solvents: a study of the antioxidant action of the extracts. *The Journal of Supercritical Fluids*.

[15] Grzanna R., Lindmark L., Frondoza C. G. (2005). Ginger-an herbal medicinal product with broad anti-inflammatory actions. *Journal of Medicinal Food*.

[16] Kamtchouing P., Mbongue Fandio G. Y., Dimo T., Jatsa H. B. (2002). Evaluation of angrogenic activity of Zingiber officinale and pentadiplandra brazzeana in male rats. *Asian Journal of Andrology*.

[17] Chaturapanich G., Chaiyakul S., Verawatnapakul V., Yimlamai T., Pholpramool C. (2008). Enhancement of aphrodisiac activity in male ratsby ethanol extract of Kaempferia parviflora and exercise training. *Andrologia*.

[18] Temkitthawon P., Hinds T. R., Beavo J. A. (2011). Kaempferia parviflora, a plant used in traditional medicine to enhance sexual performance contains large amounts of low affinity PDE5 inhibitors. *Journal of Ethnopharmacology*.

[19] Leardkamolkarn V., Tiamyuyen S., Sripanidkulchai B. O. (2009). Pharmacological activity of Kaempferia parviflora extract against human bile duct cancer cell lines. *Asian Pacific Journal of Cancer Prevention : Asian Pacific Journal of Cancer Prevention*.

[20] Tewtrakul S., Subhadhirasakul S., Karalai C., Ponglimanont C., Cheenpracha S. (2009). Anti-inflammatory effects of compounds from Kaempferia parviflora and Boesenbergia pandurata. *Food Chemistry*.

[21] Saokaew S., Wilairat P., Raktanyakan P. (2017). Clinical effects of Krachaidum (Kaempferia parviflora): a systematic review. *Journal of Evidence-Based Complementary & Alternative Medicine*.

[22] Toda K., Hitoe S., Takeda S., Shimoda H. (2016). Black ginger extract increases physical fitness performance and muscular endurance by improving inflammation and energy metabolism. *Heliyon*.

[23] Sutthanut K., Sripanidkulchai B., Yenjai C., Jay M. (2007). Simultaneous identification and quantitation of 11 flavonoid constituents in Kaempferia parviflora by gas chromatography. *Journal of Chromatography A*.

[24] Sudwan P., Saenphet K., Saenphet S., Suwansirikul S. (2006). Effect of Kaemp-feria parviflora wall ex. Baker on sexual activity of male rats and its toxicity. *Southeast Asian Journal of Tropical Medicine Public Health*.

[25] Chaturapanich G., Chaiyakul S., Verawatnapakul V., Pholpramool C. (2008). Effects of Kaempferia parviflora extracts on reproductive parameters and spermatic blood flow in male rats. *Reproduction*.

[26] Trisomboon H., Tohei A., Malaivijitnond S., Watanabe G., Taya K. (2008). Oral administration of Kaempferia parviflora did not disturb male reproduction in rats. *Journal of Reproduction and Development*.

[27] Yoshino S., Kim M., Awa R., Kuwahara H., Kano Y., Kawada T. (2014). Kaempferia parviflora extract increases energy consumption through activation of BAT in mice. *Food Science & Nutrition*.

[28] Lert‐Amornpat T., Maketon C., Fungfuang W. (2017). Effect of Kaempferia parviflora on sexual performance in streptozotocin‐induced diabetic male rats. *Andrologia*.

[29] Somphol N., Hongkhuntod P., Vongpralab T., Sanchaisuriya P., Chinchiyanont W., Prasuk Y. Effect of Boesenbergia pandurata (black rhizome) supplementation on semen characteristics in male rabbits.

[30] Jitjaingam A., Kakaew A., Saenphet K., Saenphet S., Aritajat S. Effects of Kaempferia parviflora Wall. Ex. Baker on reproductive organs hematology and kidney function of male rats.

[31] Sasaki Y., Goto H., Tohda C. (2003). Effects of curcuma drugs on vasomotion in isolated rat aorta. *Biological and Pharmaceutical Bulletin*.

[32] Itthipanichpong C., Ruangrunsri N., Kemsri W., Sawasdipanich A. (2003). Antispasmotic effects of curcuminoids on isolated Guinea-pig ileum and rat uterus. *Journal of the Medical Association of Thailand*.

[33] Goto H., Sasaki Y., Fushimi H., Shibahara N., Shimada Y., Komatsu K. (2005). Effect of curcuma herbs on vasomotion and hemorheology in spontaneously hypertensive rat. *The American Journal of Chinese Medicine*.

[34] Chaturapanich G., Chaiyakul S., Verawatnapakul V., Yimlamai T., Pholpramool C. (2012). Enhancement of aphrodisiac activity in male rats by ethanol extract of Kaempferia parviflora and exercise training. *Andrologia*.

[35] Bai Y., Zhang L., Jiang Y. (2017). Identification and functional verification of MicroRNAs in the obese rat with erectile dysfunction. *Sexual Medicine*.

[36] Pan F., Xu J., Zhang Q. (2014). Identification and characterization of the MicroRNA profile in aging rats with erectile dysfunction. *The Journal of Sexual Medicine*.

[37] Barbery C. E., Celigoj F. A., Turner S. D. (2015). Alterations in microRNA expression in a murine model of diet‐induced vasculogenic erectile dysfunction. *The Journal of Sexual Medicine*.

[38] Le Bot N. (2012). miRNAs and cell-cycle control in ESCs. *Nature Cell Biology*.

[39] Cheng A. M., Byrom M. W., Shelton J., Ford L. P. (2005). Antisense inhibition of human miRNAs and indications for an involvement of miRNA in cell growth and apoptosis. *Nucleic Acids Research*.

[40] Dennis L. M. (2008). *MicroRNAs in Early Embryonic Development: Dissecting the Role of miR-290 through miR-295 in the Mouse*.

[41] Selbach M., Schwanhäusser B., Thierfelder N., Fang Z., Khanin R., Rajewsky N. (2008). Widespread changes in protein synthesis induced by microRNAs. *Nature*.

[42] Kedde M., Agami R. (2008). Interplay between microRNAs and RNA-binding proteins determines developmental processes. *Cell Cycle*.

[43] Sendler E., Johnson G. D., Mao S. (2013). Stability, delivery and functions of human sperm RNAs at fertilization. *Nucleic Acids Research*.

[44] Pratt S. L., Calcatera S. M. (2016). Expression of microRNA in male reproductive tissues and their role in male fertility. *Reproduction, Fertility, and Development*.

[45] He Z., Kokkinaki M., Pant D., Gallicano G. I., Dym M. (2009). Small RNA molecules in the regulation of spermatogenesis. *Reproduction*.

[46] Rajender S., Meador C., Agarwal A. (2012). Small RNA in spermatogenesis and male infertility. *Frontiers in bioscience (Scholar edition)*.

[47] Yadav R. P., Kotaja N. (2013). Small RNAs in spermatogenesis. *Molecular and Cellular Endocrinology*.

[48] Toloubeydokhti T., Bukulmez O., Chegini N. (2009). Potential regulatory functions of MicroRNA in the ovary. *Endocrinology*.

[49] Galliano D., Pellicer A. (2014). MicroRNA and implantation. *Fertility and Sterility*.

[50] Barad O., Meiri E., Avniel A. (2004). MicroRNA expression detected by oligonucleotide microarrays: system establishment and expression profiling in human tissues. *Genome Research*.

[51] Liang Y., Ridzon D., Wong L., Chen C. (2007). Characterization of microRNA expression profiles in normal human tissues. *BMC Genomics*.

[52] Wu W., Hu Z., Qin Y. (2012). Seminal plasma microRNAs: potential biomarkers for spermatogenesis status. *MHR: Basic science of reproductive medicine*.

[53] McIver S. C., Roman S. D., Nixon B., McLaughlin E. A. (2012). miRNA and mammalian male germ cells. *Human Reproduction Update*.

[54] Bouhallier F., Allioli N., Lavial F. (2010). Role of miR-34c microRNA in the late steps of spermatogenesis. *RNA*.

[55] Yang Q.-E., Racicot K. E., Kaucher A. V., Oatley M. J., Oatley J. M. (2013). MicroRNAs 221 and 222 regulate the undifferentiated state in mammalian male germ cells. *Development*.

[56] Hatzfeld J., Li M. L., Brown E. L. (1991). Release of early human hematopoietic progenitors from quiescence by antisense transforming growth factor beta 1 or Rb oligonucleotides. *Journal of Experimental Medicine*.

[57] Wu J., Bao J., Kim M., Yuan S., Tang C., Zheng H. (2014). Two miRNA clusters, miR-34b/c and miR-449, are essential for normal brain development, motile ciliogenesis, and spermatogenesis. *Proceedings of the National Academy of Sciences*.

[58] Wang C.-H., Lee D. Y., Deng Z. (2008). MicroRNA miR-328 regulates zonation morphogenesis by targeting CD44 expression. *PLoS One*.

[59] Momeni A., Najafipour R., Hamta A., Jahani S., Moghbelinejad S. (2020). Expression and methylation pattern of hsa-miR-34 family in sperm samples of infertile men. *Reproductive Sciences*.

[60] Liu W.-M., Pang R. T. K., Chiu P. C. N. (2012). Sperm-borne microRNA-34c is required for the first cleavage division in mouse. *Proceedings of the National Academy of Sciences*.

[61] Comazzetto S., Di Giacomo M., Rasmussen K. D. (2014). Oligoasthenoteratozoospermia and infertility in mice deficient for miR-34b/c and miR-449 loci. *PLoS Genetics*.

[62] Jones P. A. (2012). Functions of DNA methylation: islands, start sites, gene bodies and beyond. *Nature Reviews Genetics*.

[63] Yafi F. A., Jenkins L., Albersen M. (2016). Erectile dysfunction. *Nature Reviews Disease Primers*.

[64] Li C., Li X., Gao X. (2014). MicroRNA-328 as a regulator of cardiac hypertrophy. *International Journal of Cardiology*.

[65] Fungfuang W., Lert-Amornpat T., Maketon C. (2016). Effects of Black ginger (Kaempferia parviflora) on the testicular function in streptozotocin-induced diabetic male rats. *Veterinary Integrative Sciences*.

[66] Baba S. A., Malik S. A. (2015). Determination of total phenolic and flavonoid content, antimicrobial and antioxidant activity of a root extract of Arisaema jacquemontii Blume. *Journal of Taibah University for Science*.

[67] Du W., Liang H., Gao X. (2016). MicroRNA-328, a potential anti-fibrotic target in cardiac interstitial fibrosis. *Cellular Physiology and Biochemistry*.

[68] Kaur C., Kapoor H. C. (2002). Anti-oxidant activity and total phenolic content of some Asian vegetables. *International Journal of Food Science and Technology*.

[69] McDonald S., Prenzler P. D., Antolovich M., Robards K. (2001). Phenolic content and antioxidant activity of olive extracts. *Food Chemistry*.

[70] Chang C., Yang M., Wen H., Chern J. (2002). Estimation of total flavonoid content in propolis by two complementary colorimetric methods. *Journal of Food and Drug Analysis*.

[71] Villaño D., Fernández-Pachón M. S., Moyá M. L., Troncoso A. M., García-Parrilla M. C. (2007). Radical scavenging ability of polyphenolic compounds towards DPPH free radical. *Talanta*.

[72] Vyas D., Kumar S. (2005). Purification and partial characterization of a low temperature responsive Mn-SOD from tea (Camellia sinensis (L.) O. Kuntze). *Biochemical and Biophysical Research Communications*.

[73] Zhao H., Fan W., Dong J. (2008). Evaluation of antioxidant activities and total phenolic contents of typical malting barley varieties. *Food Chemistry*.

[74] Organization for Economic Cooperation and Development (OECD) (1999). *OECD Guidelines for Testing of Chemicals. Acute Oral Toxicity*.

[75] Zodape G. V., Gaikwad V. S. (2019). Effect of Piper nigrum (linn.) on infertility induced by ethionamide and para amino salicylic acid in male sprague–dawley rats. *International Journal of Pharmaceutical Sciences and Research*.

[76] Gabr S. A., Alghadir A. H., Ghoniem G. A. (2019). Biological activities of ginger against cadmium-induced renal toxicity. *Saudi Journal of Biological Sciences*.

[77] Raji Y., Udoh U. S., Mewoyeka O. O., Okonye F. C., Bolarinwa A. F. (2003). Implication of reproductive endocrine malfunction in male infertility efficacy of Azadirahta indica extract in rats. *African Journal of Medicine & Medical Sciences*.

[78] Björndahl L., Söderlund I., Kvist U. (2003). Evaluation of the one-step eosin-nigrosin staining technique for human sperm vitality assessment. *Human Reproduction*.

[79] Khaki A., Khaki A., Hajhosseini L., Golzar F., Ainehchi N. (2014). The anti-oxidant effects of ginger and cinnamon on spermatogenesis dys-function of diabetes rats. *African Journal of Traditional, Complementary and Alternative Medicines*.

[80] Al-Rawaf H. A., Alghadir A. H., Gabr S. A. (2019). MicroRNAs as biomarkers of pain intensity in patients with chronic fatigue syndrome. *Pain Practice*.

[81] Safari F., Hosseini H., Bayat M., Ranjbar A. (2019). Synthesis and evaluation of antimicrobial activity, cytotoxic and pro-apoptotic effects of novel spiro-4H-pyran derivatives. *RSC Advances*.

[82] Chen Z.-Z., Zhang X.-D., Chen Y., Wu Y.-B. (2017). The role of circulating miR-146a in patients with rheumatoid arthritis treated by Tripterygium Wilfordii Hook. *Medicine*.

[83] Zhang M., Pan D. R., Zhou F. (2011). BP neural network extraction process by orthogonal beautiful azalea favonoids. *Journal of Xinyang Normal University*.

[84] Chivapat S., Chavalittumrong P., Attawish A., Rungsipipat A. (2010). Chronic toxicity study of Kaempferia parvifora wall ex. extract. *Tai Journal of Veterinary Medicine*.

[85] Sudwan P., Saenphet K., Saenphet S., Suwansirikul S. (2006). Efect of Kaempferia parvifora Wall. ex. Baker on sexual activity of male rats and its toxicity. *Te Southeast Asian Journal of Tropical Medicine and Public Health*.

[86] Jacob J., Amalraj A., Divya C., Janadri S., Manjunatha P. M., Gopi S. (2018). Oral toxicity study of sports nutritional powder in Wistar rats: a 90 day repeated dose study. *Toxicology Reports*.

[87] Geetha S., Sai-Ram M., Mongia S. S. (2003). Evaluation of antioxidant activity of leaf extract of sea buckthorn (*Hippophae rhamnoides* L.) on chromium (VI)induced oxidative stress in albino rats. *Journal of Ethnopharmacology*.

[88] Shimoi K., Masuda S., Shen B., Furugori M., Kinae N. (1996). Radioprotective effects of antioxidative plant flavonoids in mice. *Mutation Research: Fundamental and Molecular Mechanisms of Mutagenesis*.

[89] Shukla S., Mehta A., Bajpai V. K., Shukla S. (2009). In vitro antioxidant activity and total phenolic content of ethanolic leaf extract of Stevia rebaudiana Bert. *Food and Chemical Toxicology*.

[90] Soobrattee M. A., Neergheen V. S., Luximon-Ramma A., Aruoma O. I., Bahorun T. (2005). Phenolics as potential antioxidant therapeutic agents: mechanism and actions. *Mutation Research: Fundamental and Molecular Mechanisms of Mutagenesis*.

[91] Bravo L. (1998). Polyphenols: chemistry, dietary sources, metabolism, and nutritional significance. *Nutrition Reviews*.

[92] Agati G., Azzarello E., Pollastri S., Tattini M. (2012). Flavonoids as antioxidants in plants: location and functional significance. *Plant Science*.

[93] Chaturapanich G., Chaiyakul S., Verawatnapakul V., Yimlamai T., Pholpramool C. (2011). Enhancement of aphrodisiac activity in male rats by ethanol extract of *Kaempferia parviflora* and exercise training. *Andrologia*.

[94] Scarano W. R., Messias A. G., Oliva S. U., Klinefelter G. R., Kempinas W. G. (2006). Sexual behaviour, sperm quantity and quality after short-term streptozotocin-induced hyperglycaemia in rats. *International Journal of Andrology*.

[95] Steger R. W., Amador A., Lam E., Rathert J., Weis J., Smith M. S. (1989). Streptozotocin-induced deficits in sex behavior and neuroendocrine function in male rats. *Endocrinology*.

[96] Khawar M. B., Mehmood R., Roohi N. (2019). MicroRNAs: recent insights towards their role in male infertility and reproductive cancers. *Bosnian Journal of Basic Medical Sciences*.

[97] Pratt S. L., Calcatera S. M. (2016). Expression of microRNA in male reproductive tissues and their role in male fertility. *Reproduction, Fertility, and Development*.

[98] Marks G. S., Brien J. F., Nakatsu K. (2003). What role does the heme- heme oxygenase-carbon monoxide system play in vasoregulation?. *American Journal of Physiology-Regulatory, Integrative and Comparative Physiology*.

[99] Aziz M. T., Al-Asmar M. F., Mostafa T. (2007). Assessment of heme oxygenase-1 (HO-1) activity in the cavernous tissues of sildenafil citrate-treated rats. *Asian Journal of Andrology*.

[100] Bai Y., Jones P. P., Guo J. (2013). Phospholamban knockout breaks arrhythmogenic Ca^2+^ waves and suppresses catecholaminergic polymorphic ventricular tachycardia in mice. *Circulation Research*.

[101] Wu W., Qin Y., Li Z. (2013). Genome-wide microRNA expression profiling in idiopathic non-obstructive azoospermia: significant up-regulation of miR-141, miR-429 and miR-7-1-3p. *Human Reproduction*.

[102] Abu-Halima M., Hammadeh M., Backes C. (2014). Panel of five microRNAs as potential biomarkers for the diagnosis and assessment of male infertility. *Fertility and Sterility*.

[103] Abu-Halima M., Hammadeh M., Schmitt J. (2013). Altered microRNA expression profiles of human spermatozoa in patients with different spermatogenic impairments. *Fertility and Sterility*.

[104] Bandiera S., Hatem E., Lyonnet S., Henrion-Caude A. (2010). microRNAs in diseases: from candidate to modifier genes. *Clinical Genetics*.

[105] Hanson E. K., Lubenow H., Ballantyne J. (2009). Identification of forensically relevant body fluids using a panel of differentially expressed microRNAs. *Analytical Biochemistry*.

[106] Hayashi K., Chuva de Sousa Lopes S. M., Kaneda M. (2008). MicroRNA biogenesis is required for mouse primordial germ cell development and spermatogenesis. *PLoS One*.

[107] Lian J., Zhang X., Tian H. (2009). Altered microRNA expression in patients with non-obstructive azoospermia. *Reproductive Biology and Endocrinology*.

[108] Liu M., Liu P., Zhang L. (2011). mir-35 is involved in intestine cell G1/S transition and germ cell proliferation in C. elegans. *Cell Research*.

[109] Jung Y. H., Gupta M. K., Shin J. Y., Uhm S. J., Lee H. T. (2010). MicroRNA signature in testes-derived male germ-line stem cells. *MHR: Basic Science of Reproductive Medicine*.

[110] Niu Z., Goodyear S. M., Rao S. (2011). MicroRNA-21 regulates the self-renewal of mouse spermatogonial stem cells. *Proceedings of the National Academy of Sciences*.

[111] McIver S. C., Roman S. D., Nixon B., McLaughlin E. A. (2012). miRNA and mammalian male germ cells. *Human Reproduction Update*.

[112] Abu-Halima M., Hammadeh M., Backes C. (2014). Panel of five microRNAs as potential biomarkers for the diagnosis and assessment of male infertility. *Fertility and Sterility*.

[113] Abu-Halima M., Backes C., Leidinger P. (2014). MicroRNA expression profiles in human testicular tissues of infertile men with different histopathologic patterns. *Fertility and Sterility*.

[114] Abu-Halima M., Hammadeh M., Schmitt J. (2013). Altered microRNA expression profiles of human spermatozoa in patients with different spermatogenic impairments. *Fertility and Sterility*.

[115] Wang C., Yang C., Chen X. (2011). Altered profile of seminal plasma microRNAs in the molecular diagnosis of male infertility. *Clinical Chemistry*.

[116] Hammadeh M., Hamad M., Montenarh M., Fischer-Hammadeh C. (2010). Protamine contents and P1/P2 ratio in human spermatozoa from smokers and non-smokers. *Human Reproduction*.

[117] Ribas-Maynou J., García-Peiró A., Fernández-Encinas A. (2013). Comprehensive analysis of sperm DNA fragmentation by five different assays: TUNEL assay, SCSA, SCD test and alkaline and neutral comet assay. *Andrology*.

[118] Sharma R. K., Sabanegh E., Mahfouz R., Gupta S., Thiyagarajan A., Agarwal A. (2010). TUNEL as a test for sperm DNA damage in the evaluation of male infertility. *Urology*.

[119] Weng S.-L., Taylor S. L., Morshedi M., Schuffner A., Duran E. H., Beebe S. (2002). Caspase activity and apoptotic markers in ejaculated human sperm. *Molecular Human Reproduction*.

[120] Aitken R. J., De Iuliis G. N. (2007). Origins and consequences of DNA damage in male germ cells. *Reproductive BioMedicine Online*.

[121] Singh N. P., Muller C. H., Berger R. E. (2003). Effects of age on DNA double-strand breaks and apoptosis in human sperm. *Fertility and Sterility*.

[122] Ahmad L., Jalali S., Shami S. A., Akram Z. (2007). Sperm preparation: DNA damage by comet assay in normo- and teratozoospermics. *Archives of Andrology*.

[123] Blanco-Rodriguez J. (2002). DNA replication and germ cell apoptosis during spermatogenesis in the cat. *Journal of Andrology*.

[124] Blanco-Rodriguez J., Martinez-Garcia C. (1998). Apoptosis pattern elicited by several apoptogenic agents on the seminiferous epithelium of the adult rat testis. *Journal of Andrology*.

